# Minimal Requirements for Cancer Initiation: A Comparative Consideration of Three Prototypes of Human Leukemia

**DOI:** 10.3390/cancers16173109

**Published:** 2024-09-09

**Authors:** Toshiyuki Hori

**Affiliations:** Department of Biomedical Sciences, College of Life Sciences, Ritsumeikan University, Kusatsu 525-8577, Japan; toshihor@sk.ritsumei.ac.jp

**Keywords:** cancer initiation, two-hit model, AML, APL, CML

## Abstract

**Simple Summary:**

The pathophysiology of leukemia has been studied in the most detailed manner among all human cancers. In this review, acute promyelocytic leukemia (APL), chronic myeloid leukemia (CML), and acute myeloid leukemia (AML) with RUNX1-RUNX1T1 were selected to consider minimal requirements for cancer initiation based on a simplified model.

**Abstract:**

Even if its completed form is complex, cancer originates from one or two events that happened to a single cell. A simplified model can play a role in understanding how cancer initiates at the beginning. The pathophysiology of leukemia has been studied in the most detailed manner among all human cancers. In this review, based on milestone papers and the latest research developments in hematology, acute promyelocytic leukemia (APL), chronic myeloid leukemia (CML), and acute myeloid leukemia (AML) with RUNX1-RUNX1T1 are selected to consider minimal requirements for cancer initiation. A one-hit model can be applied to the initiation of APL and CML whereas a two-hit model is more suitable to the initiation of AML with RUNX1-RUNX1T1 and other AMLs. Even in cancer cells with multiple genetic abnormalities, there must be a few mutant genes critical for the mutant clone to survive and proliferate. Such genes should be identified and characterized in each case in order to develop individualized target therapy.

## 1. Introduction

For more than one hundred years after the dawn of modern pathology in the 19^th^ century, cancer had been recognized as a disease of the aberrant proliferation and expansion of somatic cells [1]. In those days, anti-cancer therapy was mostly based on radiation or cytotoxic chemotherapy in case operative resection was not applicable. These non-surgical therapies turned out to be ineffective to some types of cancer, especially in advanced stages, implying that deeper insights into the pathogenesis of cancer would be needed.

Recent progress in cellular and molecular biology together with the use of genetically modified animals has disclosed genetic as well as phenotypic changes occurring in cancer cells in detail. Cancer is now thought to be a disease of the uncontrolled monoclonal proliferation of cells with certain genetic abnormalities. The multi-step model of cancer has been proposed and widely accepted, in which cancer develops step by step from an early to an advanced stage through the accumulation of genetic changes and cellular selection [2,3]. 

Our understanding of the overall picture of cancer including cancer cells themselves and their surrounding microenvironment has evolved into the concept of the hallmarks of cancer [4]. Currently, eight hallmarks have been listed: the acquired capabilities for sustaining proliferative signaling, evading growth suppressors, resisting cell death, enabling replicative immortality, inducing/accessing vasculature, activating invasion and metastasis, reprogramming cellular metabolism, and avoiding immune destruction [5,6].

Investigation of all these hallmarks is expected to inform us of numerous aspects of cancer, enabling us to overcome difficulties in its treatment, especially in advanced stages. However, along with the increase in the number of the hallmarks of cancer together with the multi-step model, our knowledge of cancer has become more and more complex and diversified, which might let us overlook the fundamental question regarding cancer: how it emerges. Even if its completed form is complex, cancer originates from one or two events that happened to a single cell.

A simplified model can play a role in understanding how cancer initiates at the beginning [7]. The best-known example would be the two-hit model of retinoblastoma. In 1971, A. Knudson came up with this idea based on the published case reports of this disease [8]. He hypothesized that a certain gene in retinal cone cells mutated with a constant incidence rate as they proliferate during the first several years of life, and that individuals with genetic mutations in one allele of this gene would develop the disease when the other allele mutated. This assumption was shown to match with the incidence of the disease, and the responsible gene was later identified as Rb [9].

The two-hit model of retinoblastoma remains unchallenged up to today without correction: retinoblastoma develops when two alleles of Rb are mutated or lost, and other genetic mutations are not necessary for the development of the disease [10]. Recent studies have demonstrated that uncontrolled proliferation caused by the functional loss of RB is confined only to differentiating human cone precursor cells which have higher MDM2 expression and MYCN transcription factor activity, and that it never happens in other types of human retinal cells or the cone precursor cells of other animals [11,12]. 

Although retinoblastoma may be an exceptional case among various types of cancers in that it neither originates from a stem cell nor needs multi-step progression, the elucidation of its pathogenesis has reminded us of a basic framework of thinking that is applicable to other types of cancer. It is apparent that there is a certain order of critical events necessary for cancer initiation and those events must occur during a narrow window of the differentiation stage of a limited cell lineage. In the present review, three possible scenarios of the onset of leukemia are considered from this point of view and compared with other types of cancer.

## 2. Initiation of Leukemia

The pathophysiology of leukemia has been studied in the most detailed manner among all human cancers because of the bedside availability of single cell preparations of leukemic cells. For decades, leukemia has been classified into several subtypes, based on clinical course, cell lineage, and karyotype [13]. The cellular properties and genotypes of each subtype have been analyzed and the vast resulting data have been compiled.

It has been recognized that the presence of a fusion gene created by chromosomal translocation often plays an essential role in the development of certain subtypes of leukemia rather than point mutations or the deletion of a gene. High incidence of such typical karyotypes in leukemia is now considered to be ascribed to the vicinity of the corresponding chromosomes during mitosis in hematopoietic cells [14,15]. At least two subtypes of leukemia, acute promyelocytic leukemia (APL) with the chromosomal translocation of t(15;17)(q22;21) and chronic myeloid leukemia (CML) with t(9;22)(q34;q11), are thought to develop because of only one type of genetic change like retinoblastoma. 

## 3. Acute Promyelocytic Leukemia

In 2000, M. Vickers et al. surveyed 159 cases of APL and found that except for the period of childhood, the incidence of APL is approximately constant over most of the age range and does not increase with age, suggesting that the event causing APL is restricted to cells committed to differentiation and not to stem cells and that the data were not compatible with a conventional multi-hit model of carcinogenesis [16]. This means that halfway-differentiated cells self-renew to generate cancer as the case of retinoblastoma.

APL is characterized by the presence of the t(15;17)(q22;21) chromosomal translocation that generates the PML-RARα fusion gene. The chimeric protein retains the DNA-binding and ligand-binding domains of RARα and the multimerization domain of PML. In normal cells, PML is a main constituent of nuclear bodies, which are matrix-associated multiprotein-containing domains involved in various biological functions. The presence of PML-RARα hinders the localization of the wild-type PML from nuclear bodies to numerous micro speckles and induces a maturation block at the promyelocytic stage [17]. It should be noted that PML-RARα itself does not trigger signals for proliferation.

According to the recent report of four APL cases with blood DNA available before diagnosis, highly sensitive PCR identified none of the PML-RARα fusions in their relevant pre-leukemic samples, which supports the previous conjecture that a single event can initiate leukemia within a short period of time [18].

**Figure 1:**
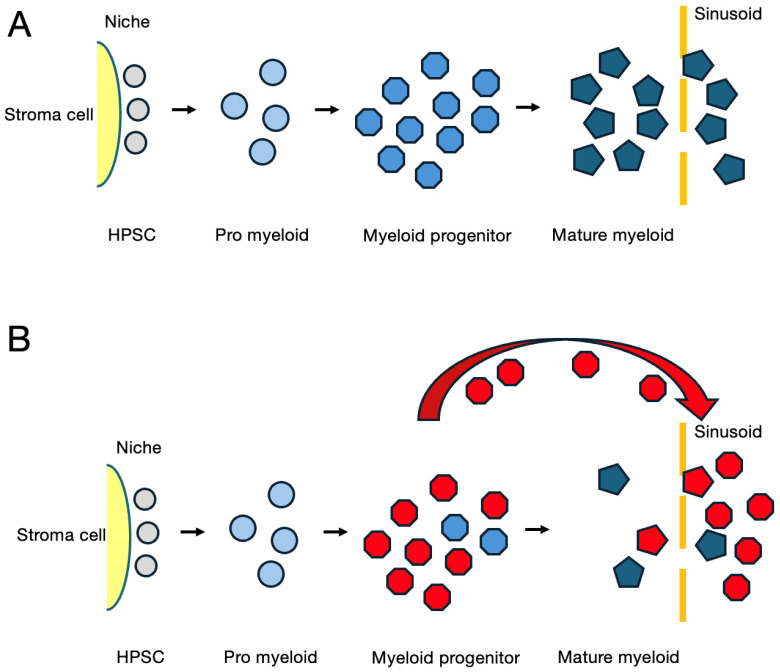
(**A**) A model of a normal bone marrow showing the myeloid maturation pathway. HPSC indicated by grey circles are located close to a stroma cell in niche. (**B**) A model of APL. Red cells represent the mutant clone.

A scheme of normal myeloid hematopoiesis in the bone marrow and a model of leukemogenesis of APL are depicted in Figure 1A and Figure 1B, respectively. Hematopoietic stem cells (HPSCs) adjacent to the niche are not affected in APL. The first hit at the stage of promyelocyte or its near precursor resulted in a defect of further differentiation. The APL clone continues its proliferation in response to cytokines such as G-SCF and GM-SCF present in the bone marrow microenvironment. An excess number of leukemic cells overflow to the sinusoid and then to the periphery. The point here is that the formation of the fusion gene alone is sufficient for the initiation of leukemia. Cellular proliferation is mostly paracrine -factor dependent and is ascribed to the maturation arrest.

## 4. Leukemia Stem Cell

Before consideration of the early events in other subtypes of leukemia, the concept of leukemia stem cell as well as normal stem cell needs to be addressed. It is well known that normal hematopoiesis is a hierarchy in which solely stem cells located in the bone marrow niche have the capacity of both self-renewal and production of multi-lineage progeny [19]. In 1997, T. Lapidot et al. refined the definition of the leukemia stem cell as the cell capable of initiating human AML in NOD/SCID mice and showed that those cells possessed differentiative as well as proliferative capacities and the potential for self-renewal [20]. They found the presence of such cells in the primitive CD34^+^ CD38^-^ but not in the more mature CD34^+^ CD38^+^ fraction, suggesting that the leukemia clone is also organized as a hierarchy [21].

Since then, in vivo reproducibility of human leukemia has become the certificate for the leukemia stem cell and until now several modified model mice designed to have no NK cells and express high amounts of hematopoietic cytokines have been utilized to characterize leukemia stem cells. These xenografts have proved a better engraftment of human AML and yielded more heterogeneity in leukemia stem cell populations including CD34^+^ CD38^+^ cell populations, suggesting the critical importance of the microenvironment in vivo [22,23].

In accordance with this, recent evidence has indicated that hematopoietic cell fates are not as rigidly encoded in stem cells and progenitors as previously envisioned and that interpretations of experiments involving stem cells are profoundly influenced by the assay used for analysis [24].

By definition, a normal as well as a leukemia stem cell must have the property of self-renewal. However, it is now clear that the leukemia stem cell does not have to be undifferentiated, and its clone does not have to be organized as a hierarchy in contrast to a normal stem cell. As described above, a differentiated cell can become a leukemia stem cell in APL just as in the case of retinoblastoma.

Another thing to be mentioned here is the stroma cells in the bone marrow niche. Their real nature remains largely unknown but accumulating evidence has indicated that most of them are of mesenchymal origin. They are speculated to support the self-renewal of hematopoietic stem cells through direct cell-to-cell interaction [25]. As to the functional cell mapping of the human bone marrow niche, S. Bandyopadhyay et al. have recently indicated that adipocytes and THY1+ mesenchymal cells co-localize with primitive hematopoietic cells and express most supportive factors through analysis of human bone marrow cells by single-cell RNA sequencing combined with co-detection by indexing, and that this type of stroma cell is not present in murine bone marrow [26]. The latter point may have some relevance to the results of engraftment in xenograft assays.

## 5. Chronic Myeloid Leukemia

CML is a clonal myeloproliferative disorder caused by t(9;22)(q34;q11) in a hematopoietic stem cell [27]. In its typical form, the translocation generates BCR-ABL1, a 210 KD chimeric protein, which results in expansion of the leukemia clone. A statistical analysis of 5256 CML cases observed between 1973 and 2002 indicated that the incidence of chronic phase CML per year is consistent with the hypothesis that the chromosomal translocation alone is sufficient to cause the disease, namely, the one-hit model [28].

As normal stem cells, CML stem cells have been shown to be dependent on unspecified signals provided through direct interaction with stromal cells in the bone marrow niche. β-catenin/Wnt signals may be involved in this process because normal as well as CML stem cells of β-catenin-deficient mice have been reported to show impaired self-renewal potential [29]. BCR-ABL1 does not cause maturation arrest and CML stem cells differentiate, exit the niche, and mature to myeloid progenitor cells responsive to G-SCF and GM-SCF. The leukemia clone rapidly proliferates in the presence of these cytokines because BCR-ABL1 serves as a platform for Jak2 associated with the cytokine receptors and facilitates its function to activate the downstream signaling through the Ras and PI3 kinase pathway [30]. 

BCR-ABL1 is a constitutively active tyrosine kinase and is counted as an oncogene product. However, it cannot induce proliferation by itself and IL-3 receptor/Jak2/Stat5 pathways are required for BCR-ABL1 to induce the oncogenic transformation of NIH 3T3 fibroblasts [31]. This is in accordance with the case of another oncogenic tyrosine kinase, v-Src, in that its expression induces chromosomal abnormalities and p21-mediated cell cycle arrest. The delayed formation of transformed colonies by v-Src is attributed to chromosomal abnormalities through stochastic genetic alterations, but not the continuous stimulation of growth signaling [32]. Thus, strong signals of oncogenic tyrosine kinases by themselves do not initiate cancer as previously thought.

**Figure 2:**
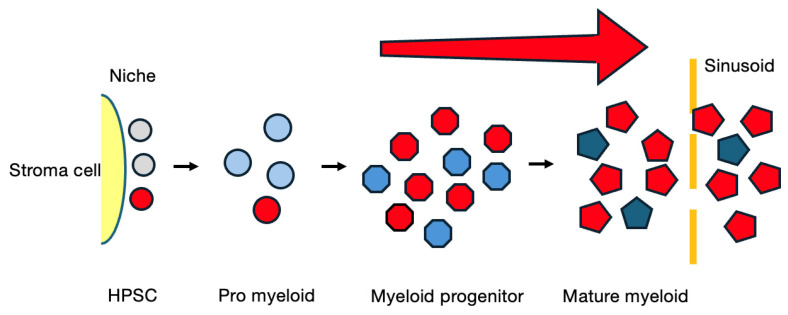
A model of chronic phase CML. Red cells represent the mutant clone.

Figure 2 shows a simplified model of the cellular flow in bone marrow in the chronic phase of CML. BCR-ABL1 does not hinder maturation but just augments the proliferative response of the CML clone to cytokines at a limited stage during myeloid maturation. Therefore, if the first hit occurred at the myeloid progenitor cell level as in the case of APL, the BCR-ABL1^+^ clone would be pushed out by normal stem cell-derived myeloid cells from the bone marrow sooner or later. In other words, this event must occur in a hematopoietic stem cell with self-renewal potential in order to initiate leukemia. Other chronic myeloproliferative disorders such as polycythemia vera and essential thrombocythemia, which are both caused by cytokine receptor abnormalities, are supposed to be categorized in this prototype [33].

## 6. Acute Myeloid Leukemia with RUNX1-RUNX1T1

Several recurrent chromosomal abnormalities and gene mutations have been detected and listed in leukemia. Among them, t(8;21)(q22;q22.1) is one of the most frequent chromosomal translocations and AML with this karyotype has been investigated intensively since its discovery in 1973 [34,35]. Advanced sequencing technology and molecular cell biology have revealed that t(8;21)(q22;q22.1) generates the fusion gene of RUNX1-RUNX1T1 and that the presence of RUNX1-RUNX1T1 causes the recruitment of repressors of transcription to blocking the expression of genes involved in hematopoietic cell maturation [36].

**Figure 3:**
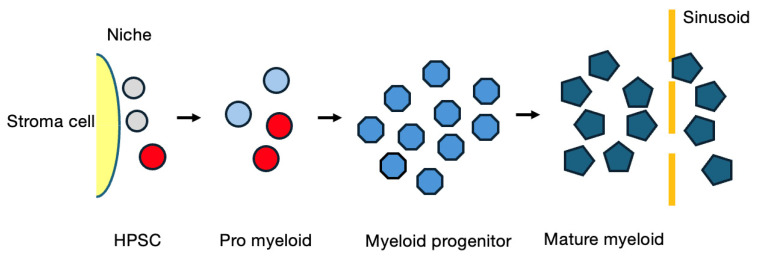
A model of the pre-leukemia stage of AML. Red cells represent the mutant clone.

For an unknown reason, this event seems to fit the intrinsic properties of stem cells or very immature myeloid cells close to stem cells and the resultant mutant clone would stay in niche for a long period of time, which is supported by a report that AML1-ETO (RUNX1-RUNX1T1) transcripts could be detected in Guthrie cards from newborns who develop AML later in life [37]. Furthermore, chronological analysis of the clonal composition of AML samples has revealed that RUNX1-RUNX1T1 is present early in the preleukemic clone, is also found in clones when patients relapse, and leads to nonleukemic hematopoiesis in the marrows of mice in xenotransplantation models [38]. Experiments with transgenic mice have shown that RUNX1- RUNX1T1 knock-in led to the expansion of myeloid progenitor cells but did not block their differentiation nor initiate leukemia [39,40]. Thus, RUNX1-RUNX1T1 can be the first hit but is not sufficient for the initiation of leukemia as depicted in Figure 3.

Additional genetic abnormalities in combination with RUNX1-RUNX1T1 have been investigated as follows. M.-T. Krauth et al. performed a comprehensive genetic analysis of 139 AML patients with RUNX1-RUNX1T1 and found that the most frequent coexisting mutations were KIT, followed by NRAS, ASXL1 FLT3-ITD, FLT3-TKD, CBL, and KRAS, and overall RAS pathway-activating mutations including NRAS, KRAS, FLT3-ITD, FLT3-TKD, CBL, and JAK2 were found in 30.9% [41]. N. Duployez et al. reported that 96% of 106 patient with t(8;21)(q22;q22.1) carried additional cytogenetic or mutational anomalies such as KIT, FLT3-ITD, and RAS [42].

**Figure 4:**
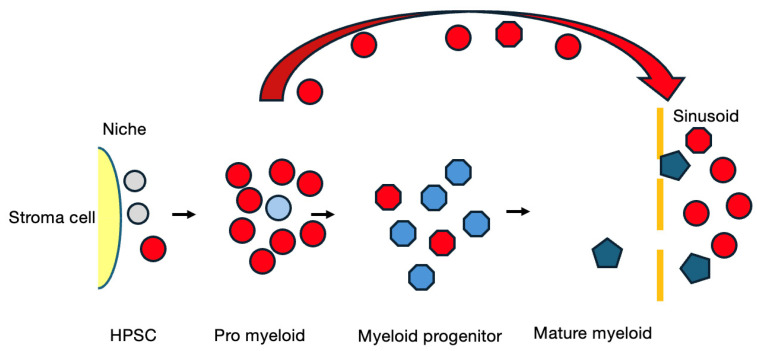
A model of the overt stage of AML. Red cells represent the mutant clone.

These results suggest that the presence of the RUNX1-RUNX1T1 blocks differentiation and promotes self-renewal of an immature cell but is not sufficient to initiate leukemia and a second hit of the genetic event that is related to cytokine signaling is needed. Based on this assumption, a possible scenario of this subtype of AML is depicted in Figure 4.

According to an extensive survey of 1540 patients with AML published in 2014, among 5234 driver mutations across 76 genes or genomic regions, it was found that most patients had more than two driver mutations and frequent mutations were NPM1, DNMT3A, FLT3ITD, NRAS, TET2, and PTPN11 in this order [43]. NPM1, DNMT3A, and TET2 are known to be involved in chromatin remodeling and cell differentiation while FLT3ITD, NRAS, and PTPN11 function in cytokine receptor-related signaling.

We do not have enough data to understand the whole picture of AML pathogenesis as yet. The roles of most of the genes involved in epigenetics remain to be elucidated. However, accumulating evidence has suggested that at least two genetic changes of different functional categories are required. As shown in this prototype, the first hit is likely to be a genetic change inducing self-renewal and maturation arrest which can lead to a preleukemia state. The second hit in most cases may be a genetic change related to cytokine receptor-mediated signaling. 

## 7. Comparison with Retinoblastoma and Colorectal Cancer

Genetic mutation is prone to occur during DNA replication. Therefore, tissues with rapid cell turnover such as skin, mucosal membrane, and bone marrow have more chance of generating cancer than static tissues such as muscle and the neuronal system.

Retinal pigment epithelium is an exception because cone precursor cells are programmed to divide regularly during the first several years of life as a part of development without the premise of mutation. Human cone precursor cells have been found to be specifically sensitive to Rb loss because of the increased expression of MDM2, MYCN, and other proliferation-related proteins [11,12]. Once the mutant clone begins to grow and mature, the clonal cells will never exfoliate out of the body as epithelial cells of other tissues facing out of the body because an eyeball is an internal enclosed space. Normal cone cells replicate only a limited number of times and are never replaced or replenished. These tissue-specific features apparently contribute to the onset of the disease.

**Figure 5:**
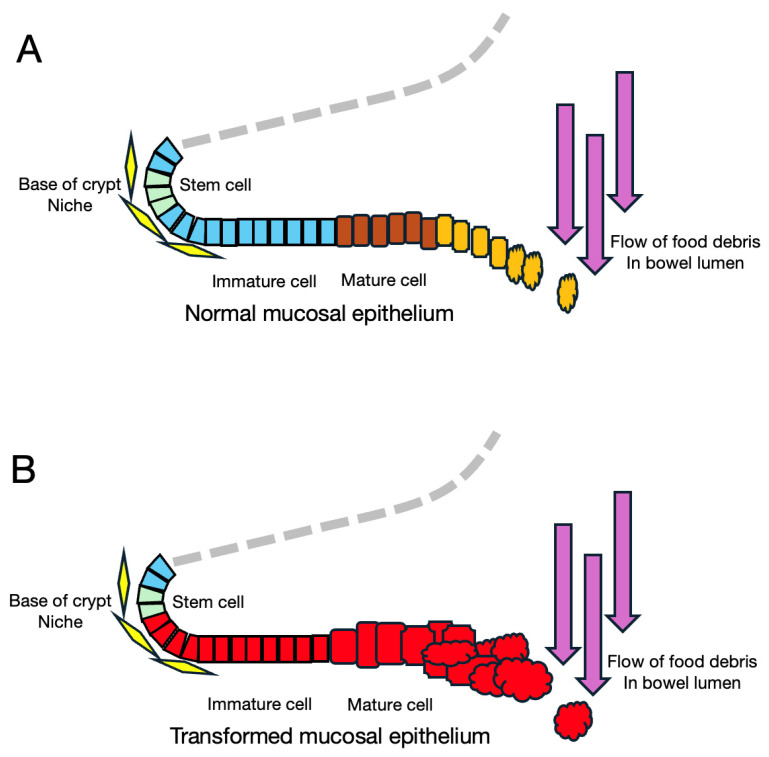
(**A**) A scheme of a normal colon crypt. Stem cells at the bottom of the crypt give rise to progeny of epithelial cells which proliferate and mature. Terminally differentiated cells are pushed away into the bowel lumen. (**B**) Red mutant cells proliferate rapidly to form a polyp but can be pushed out in the meantime.

Colorectal epithelium is entirely different from retinal pigment epithelium in that the former is maintained by rapid cell turnover with the stem cell/niche system. Cells of the outer region of the crypt are steadily replaced by newly differentiating cells derived from stem cells staying in the niche at the bottom of the crypt as shown in Figure 5A. While stem cells repeat senescence and replication with the help of mesenchymal stroma cells, immature differentiating cells proliferate in response to Wnt signals-induced accumulation of β-catenin/TCF [44]. This stem cell/niche system works as a safeguard to prevent cancer, because resting stem cells have less chance of undergoing mutation and, even if a mutation occurs in a proliferating cell, the mutant clone will be gradually pushed out by newly supplied normal cells to the lumen of the bowel then out of the body, as shown in Figure 5B. Accordingly, the first hit must occur in a stem cell to initiate colorectal cancer.

The bone marrow has the stem cell/niche system as the colorectal epithelium does, but the anatomical structure is different in each. Bone marrow is an enclosed space filled with miscellaneous cells and does not face to outside of the body. Primitive hematopoietic cells proliferate and mature while staying in the bone marrow until the time of exit into the blood flow when they finish differentiation. In contrast to the bowel lumen, the intravascular space is not outside of the body. Blood cells including immature precursors can re-enter the bone marrow after systemic circulation. This is why a single mutation in a differentiating cell can initiate cancer (APL) in bone marrow. Presumably, further mutations enabling epithelial-mesenchymal transition are required for a colorectal polyp to become cancer. 

## 8. Additional Consideration

Most genetic abnormalities, namely the point mutation, deletion, insertion, duplication, and double strand break take place during DNA replication and the double strand break can cause aberrancy of chromosomes. Since these events are associated with cell division, it is natural to think that a proliferating cell has more chance of undergoing the first hit for cancer initiation.

A cancer cell with multiple genetic changes must have its history of serial genetic events, but it is difficult for us to determine the chronological order and the significance of events retrospectively. There are a few types of cancer or leukemia in which a single gene mutation, whether it is diploid or haploid, can initiate cancer. The detailed analyses of these types have disclosed a basic framework for cancer initiation and seem to give us hints to understand the origin of the cancer cell with multiple genetic changes after completion of the disease.

Most genetic changes are in principle harmful to a cell and a single gene mutation can be fatal, especially in the case of a gene for basic cellular metabolism or catabolism. Therefore, for a future cancer cell to stay alive and survive in competition with an overwhelming majority of normal cells in a in vivo microenvironment, the possible choices of the first hit are restricted. 

As has been discussed earlier, the first hit in most cases of AML is likely to be a genetic change inducing self-renewal and maturation arrest and this category of genetic events must occur at the very early stage of maturation or in the narrow range of a differentiated stage. Such a mutation at the stem cell level would result in the persistence of the mutant clone in the bone marrow niche with a minimal cell replication rate. A similar mutation at the promyelocyte stage would enable the mutant clone to continue proliferation in response to hemopoietic cytokines and outgrow normal counterparts. In contrast, if it occurred at the other stages without anchoring to the niche or cytokine responsiveness, the mutant clone would be pushed out of the bone marrow by normal counterpart cells and never stay long.

Chromatin organization and function which determine the cell fate must be finely tuned in hematopoietic stem cells. If a genetic mutation changed their state dramatically, then the mutant clone would have to exit the niche. Accordingly, there are limited choices for this category of genetic events and the first hit event decides the fate of the clone afterwards. The mutant clone should retain most of the characteristics of the stem cell, stay in the niche, and repeat self-renewal in the presence of the stem cell-matched cytokines such as SCF and FLT3. The presence of such preleukemia clones has been reported on various occasions. 

More than two types of genetic changes of different functional categories are known to be detected in most AML cases. Little is known about the nature of the second hit that follows the first hit causing maturation arrest. It seems to be as important as the first hit. Suppose that there is a selection in the niche, the second hit can be a mutation of a gene related to fitness to the niche and its dominance there. In this case, the mutant clone with limited or aberrant differentiation capacity would have more chance of occupying a large part of the niche space, and such a pathological state with clonal expansion in the bone marrow might be an equivalent of cancer like myelodysplastic syndrome.

For a mutant cell to start proliferation to evolve to overt leukemia, the second hit must be a mutation of a gene of an appropriate component in the growth signaling cascade. Cellular proliferation is composed of a complex and delicate series of reactions. An abrupt and inappropriate emergence of excessive signals is likely to elicit intrinsic protective reactions and cell cycle arrest as shown in the case of v-Src. Therefore, the choice of the second hit for overt leukemia is again limited. It seems that there is no other option than to mimic or hijack an intrinsic factor in the preexisting signaling pathway for cell growth.

Various types of cancer have their individual molecular pathogenesis. Nevertheless, in order to become a cancer clone, a single cell of any tissue or organ must follow the similar scenario at the beginning as discussed here for the prototypes of leukemia. Mutations of the genes affecting chromatin remodeling and cell differentiation such as NPM1, DNMT3A, and TET2 are frequently detected not only in AML but also in other types of cancer including solid tumors [45]. This category of mutations are primary candidates for the first hit if it occurs at the stem cell level or during a narrow range of differentiation stage under appropriate circumstances. As has been mentioned in the section on CML, the second category of mutations that drives cell growth can also be the first hit but is more likely to be the second hit.

## 9. Conclusions

Even in cancer cells with multiple genetic abnormalities, there must be a few genetic events critical for the mutant clone to survive and proliferate. One-hit or two-hit genetic events can initiate cancer depending on the nature of the cell of origin. Such genetic events seem to fall into two categories; the first one affects chromatin organization and function and the other one drives cell growth. Much is left for us to fully understand the mechanism of the first category. Both of them must be selected as being compatible with the differentiation stage, the microenvironment, and the nature of the tissue. Other genes can be additive or auxiliary. As a future challenge, core causative genes should be identified and characterized in each case of cancer in order to develop individualized target therapy.
